# Supplementary material for: Predictors of virological outcomes after analytical interruption of antiretroviral therapy and HTI vaccination in early treated people with HIV-1._
Source: Commun Med (Lond). 2025 Dec 8;6:11. doi: 10.1038/s43856-025-01266-y (PMC12775531; doi:10.1038/s43856-025-01266-y)
Supplement: Supplementary file 2 — Supplemental Information [file 43856_2025_1266_MOESM2_ESM.pdf]

Supplementary Material for "*Predictors of virological outcomes after analytical interruption of antiretroviral therapy and HTI vaccination in early treated people with HIV*".

Supplementary Fig. 1 | AELIX-002 and AELIX-003 trial designs and HIV-1 RNA pVL during the ATI period

Supplementary Table 1 | AELIX-002 and AELIX-003 study populations

Supplementary Table 2 | Clinical, virological and immunological correlates of ATI outcomes

Supplementary Table 3 | Univariate correlate analysis for time off ART

Supplementary Table 4 | Multivariate logistic regression analysis for time off ART

Supplementary Note | List of AELIX-002 and AELIX-003 Study Group members

Supplementary Fig. 1 | AELIX-002 and AELIX-003 trial designs and HIV-1 RNA pVL during the ATI period. a, **b**, AELIX-002 and AELIX-003 trial schedule and study visits. **c, d,** AELIX-002 and AELIX-003 individual HIV-1 pVL during the 24 weeks of ATI, shown for all placebo (blue) or vaccine (red) recipients.

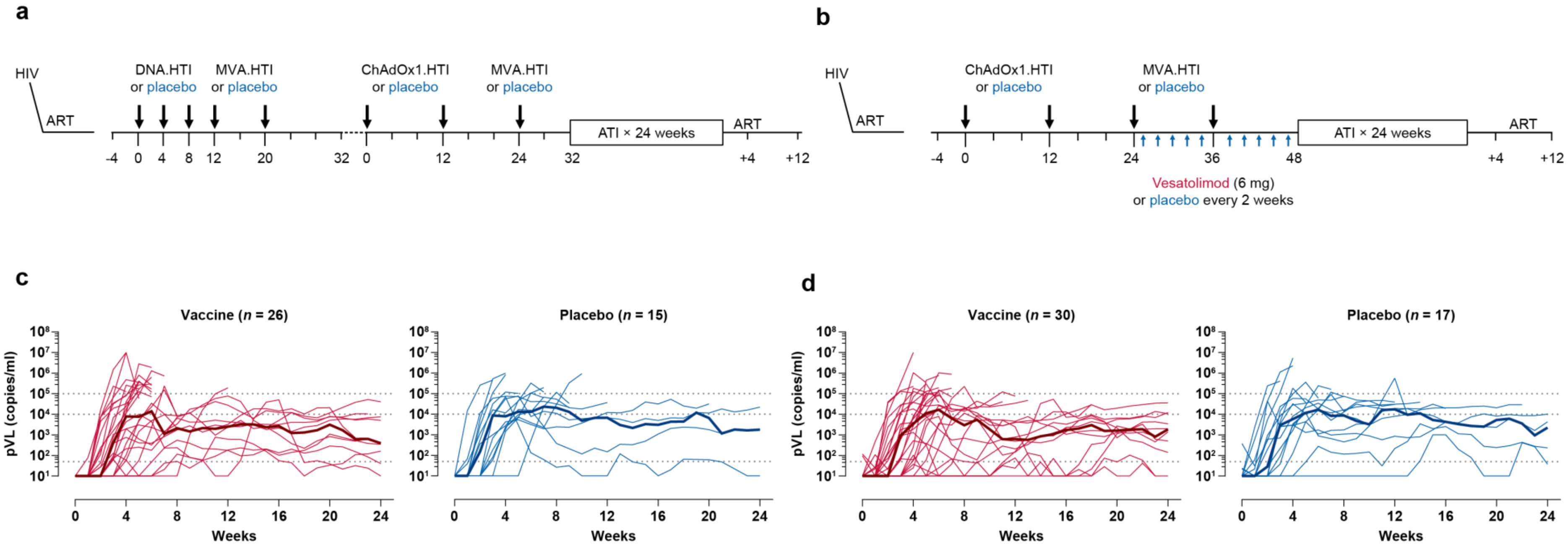

Supplementary Table 1 | AELIX-002 and AELIX-003 study populations

Main demographic and clinical characteristics of individuals included in this analysis; All participants reflect the merged AELIX-002 and AELIX-003 vaccinees and placebo recipient populationsMedian (IQR) is presented, except when it is specified differentially. Mann–Whitney test and chi-squared test used. Tests are two-sided, unadjusted for multiple comparisons, with 5% error rate.

**Characteristics**	**All participants (*n* = 88)**	**AELIX-002 (*n* = 41)**	**AELIX-003 (*n* = 47)**	***P* value**
**At study entry**				
**Treatment allocation, vaccine, *n (%)***	59 (67%)	26 (63%)	33 (70%)	0.6498
Age, years	38 (34–43)	36 (33–41)	39 (31–47)	0.5410
Sex at birth, male, *n* (%)	87 (99%)	87 (99%)	47 (100%)	0.4659
Days from estimated HIV acquisition to ART initiation	65 (30–91)	65 (31–85)	65 (28–95)	0.6190
Fiebig stage at ART initiation, *n* (%)[a]				0.2373
Eclipse	0 (0.0%)	0 (0.0%)	0 (0.0%)	
I	4 (5%)	2 (5%)	2 (4%)	
II	5 (6%)	1 (2%)	3 (6%)	
III	5 (6%)	2 (5%)	3 (6%)	
IV	7 (8%)	2 (5%)	5 (11%)	
V	39 (44%)	22 (54%)	17 (36%)	
VI	23 (26%)	12 (29%)	11 (23%)	
Missing	5 (6%)	0 (0%)	5 (11%)	
HIV-1 pVL at ART initiation ($\log_{10}$ copies/ml)	5.0 (4.4–6.0)	4.8 (4.4–5.3)	5.3 (4.4–6.2)	0.0640
Time on ART at study entry, months	35 (23–50)	28 (19–39)	44 (31–55)	<0.0010
**INSTI-based ART at study entry, *n (%)***	74 (84%)	41 (100%)	33 (70%)	<0.0001
Beneficial HLA alleles, any[b]	18 (20%)	9 (22%)	9 (19%)	0.9400
**At ATI start**				
Absolute CD4 (cells/mm^3)	863 (697–1,130)	860 (733–1,114)	906 (649–1,175)	0.7660
Time on ART, months	52 (42–64)	49 (42–58)	55 (44–67)	0.3300
Total proviral HIV-1 DNA (copies/10^6 $CD4^+$ T cells)	113 (49–294)	90 (40–261)	159 (50–437)	0.1090
Intact proviral HIV-1 DNA (copies/10^6 $CD4^+$ T cells)	29 (8–90)	14 (3–42)	58 (13–142)	0.0030
HTI magnitude (SFC/10^6 PBMC)	445 (125–995)	325 (105–840)	746 (204–1,210)	0.0650

[a]According to Fiebig, AIDS 2003. [b]Beneficial HLA class I alleles associated with spontaneous viral control (B*27:05, B*57:01, B*15:17, B*15:03, B*58:01).

Supplementary Table 2 | Clinical, virological and immunological correlates of ATI outcomes. Spearman's ρ and *P* value are shown for each correlation between baseline characteristics, viral reservoir, immune parameters and ATI outcomes by active and placebo groups, and when considering pooled data for all participants in AELIX-002 and AELIX-003. All tests are two-sided, unadjusted for multiple comparisons, with 5% error rate. *P* values < 0.05 are highlighted.

	**Placebo; *N* = 32**				**Active; *N* = 56**				**Pooled; *N* = 88**			
	**Time to pVL >50**	**Time to pVL >10K**	**Time off ART**	**pVL at end of ATI**	**Time to pVL >50**	**Time to pVL >10K**	**Time off ART**	**pVL at end of ATI**	**Time to pVL >50**	**Time to pVL >10K**	**Time off ART**	**pVL at end of ATI**
**Baseline characteristics**												
Age	ρ = 0.06<br>*P* = 0.730	ρ = 0.03<br>*P* = 0.871	ρ = 0.09<br>*P* = 0.641	ρ = 0.03<br>*P* = 0.856	ρ = 0.20<br>*P* = 0.143	ρ = 0.18<br>*P* = 0.178	ρ = 0.08<br>*P* = 0.580	ρ = 0.05<br>*P* = 0.703	ρ = 0.18<br>*P* = 0.094	ρ = 0.15<br>*P* = 0.171	ρ = 0.08<br>*P* =0.469	ρ = 0.01<br>*P* = 0.899
Absolute CD4^{+} T cells	ρ = 0.28<br>*P* =0.127	ρ = 0.25<br>*P* = 0.167	ρ = 0.10<br>*P* = 0.599	ρ = 0.07<br>*P* = 0.719	ρ = 0.00<br>*P* = 0.995	ρ = 0.16<br>*P* = 0.230	ρ = 0.23<br>*P* = 0.091	ρ =–0.22<br>*P* =0.103	ρ =0.07<br>*P* = 0.542	ρ = 0.17<br>*P* = 0.124	ρ = 0.18<br>*P* = 0.098	ρ = –0.13<br>*P* = 0.237
CD4/CD8	ρ = 0.33<br>*P* = 0.064	ρ = 0.16<br>*P* = 0.390	ρ = 0.14<br>*P* = 0.448	ρ = –0.12<br>*P* = 0.532	ρ = 0.06<br>*P* = 0.658	ρ = 0.16<br>*P* = 0.235	ρ = 0.19<br>*P* = 0.166	ρ = –0.25<br>*P* = 0.062	ρ = 0.14<br>*P* = 0.188	ρ = 0.14<br>*P* = 0.180	ρ = 0.18<br>*P* = 0.095	ρ = –0.21<br>*P* = 0.057
Pre-ART pVL	ρ = –0.23<br>*P* = 0.209	ρ = –0.26<br>*P* = 0.143	ρ = –0.22<br>*P* = 0.222	ρ = 0.11<br>*P* = 0.548	ρ = –0.44<br>*P* = **0.001**	ρ = –0.44<br>*P* = **0.001**	ρ = –0.33<br>*P* = **0.012**	ρ = 0.35<br>*P* = **0.010**	ρ = –0.34<br>*P* = **0.001**	ρ = –0.36<br>*P* < **0.001**	ρ = –0.31<br>*P* = **0.004**	ρ = 0.27<br>*P* = **0.011**
Days from HIV to ART	ρ = 0.12<br>*P* = 0.515	ρ = 0.16<br>*P* = 0.371	ρ = 0.26<br>*P* = 0.154	ρ = –0.24<br>*P* = 0.202	ρ = 0.21<br>*P* = 0.128	ρ = 0.04<br>*P* = 0.765	ρ = –0.06<br>*P* = 0.635	ρ = 0.06<br>*P* = 0.654	ρ = 0.13<br>*P* = 0.241	ρ = 0.08<br>*P* = 0.483	ρ = 0.05<br>*P* = 0.632	ρ = –0.03<br>*P* = 0.752
Time on ART	ρ = 0.18<br>*P* = 0.321	ρ = 0.06<br>*P* = 0.765	ρ = 0.12<br>*P* = 0.521	ρ = –0.18<br>*P* = 0.345	ρ = 0.32<br>*P* = **0.016**	ρ = 0.32<br>*P* = **0.017**	ρ = 0.29<br>*P* = **0.029**	ρ = –0.29<br>*P* = **0.032**	ρ = 0.29<br>*P* = **0.005**	ρ = 0.23<br>*P* = **0.031**	ρ = 0.22<br>*P* = **0.040**	ρ = –0.25<br>*P* = **0.019**
**Viral reservoir**												
Total proviral HIV-1 DNA at study entry	ρ = –0.19<br>*P* = 0.289	ρ = –0.47<br>*P* = **0.007**	ρ = –0.54<br>*P* = **0.002**	ρ = 0.35<br>*P* = 0.053	ρ = –0.05<br>*P* = 0.698	ρ = –0.26<br>*P* = 0.057	ρ = –0.14<br>*P* = 0.304	ρ = 0.23<br>*P* = 0.096	ρ = –0.09<br>*P* = 0.411	ρ = –0.34<br>*P* = **0.001**	ρ = –0.29<br>*P* = **0.006**	ρ = 0.27<br>*P* = **0.014**
Intact proviral HIV-1 DNA at study entry	ρ = –0.01<br>*P* = 0.948	ρ = –0.47<br>*P* = **0.029**	ρ = –0.64<br>*P* = **0.001**	ρ = 0.53<br>*P* = **0.013**	ρ = –0.19<br>*P* = 0.182	ρ = –0.36<br>*P* = **0.011**	ρ = –0.21<br>*P* = 0.136	ρ = 0.38<br>*P* = **0.008**	ρ = –0.12<br>*P* = 0.304	ρ = –0.40<br>*P* = **0.001**	ρ = –0.35<br>*P* = **0.002**	ρ = 0.39<br>*P* = **0.001**
Total proviral HIV-1 DNA at ATI start	ρ = –0.10<br>*P* = 0.594	ρ = –0.07<br>*P* = 0.721	ρ = –0.10<br>*P* = 0.573	ρ = 0.14<br>*P* = 0.459	ρ = –0.09<br>*P* = 0.524	ρ = –0.30<br>*P* = **0.025**	ρ = –0.18<br>*P* = 0.184	ρ = 0.22<br>*P* = 0.100	ρ = –0.08<br>*P* = 0.463	ρ = –0.21<br>*P* = **0.045**	ρ = –0.15<br>*P* = 0.171	ρ = 0.19<br>*P* = 0.088
Intact proviral HIV-1 DNA at ATI start	ρ = 0.00<br>*P* = 0.996	ρ = –0.39<br>*P* = 0.072	ρ = –0.50<br>*P* = **0.017**	ρ = 0.45<br>*P* = **0.041**	ρ = –0.15<br>*P* = 0.287	ρ = –0.33<br>*P* = **0.017**	ρ = –0.22<br>*P* = 0.122	ρ = 0.27<br>*P* = 0.057	ρ = –0.09<br>*P* = 0.429	ρ = –0.35<br>*P* = **0.003**	ρ = –0.31<br>*P* = **0.007**	ρ = 0.30<br>*P* = **0.010**
**HTI immune parameters**												
Magnitude HTI at study entry	ρ = –0.14<br>*P* = 0.479	ρ = –0.14<br>*P* = 0.475	ρ = 0.09<br>*P* = 0.647	ρ = –0.22<br>*P* = 0.267	ρ = 0.41<br>*P* = **0.002**	ρ = 0.26<br>*P* = 0.065	ρ = 0.27<br>*P* = 0.053	ρ = –0.23<br>*P* = 0.106	ρ = 0.26<br>*P* = 0.020	ρ = 0.15<br>*P* = 0.190	ρ = 0.22<br>*P* = 0.051	ρ = –0.22<br>*P* = 0.054
Focus HTI at study entry	ρ = –0.24<br>*P* = 0.227	ρ = –0.10<br>*P* = 0.603	ρ = 0.17<br>*P* = 0.401	ρ = –0.22<br>*P* = 0.275	ρ = 0.38<br>*P* = **0.005**	ρ = 0.14<br>*P* = 0.330	ρ = 0.11<br>*P* = 0.422	ρ = –0.10<br>*P* = 0.463	ρ = 0.19<br>*P* = 0.083	ρ = 0.08<br>*P* = 0.460	ρ = 0.14<br>*P* = 0.211	ρ = –0.14<br>*P* = 0.227
Breadth HTI at study entry	ρ = –0.16<br>*P* = 0.399	ρ = –0.02<br>*P* = 0.938	ρ = 0.06<br>*P* = 0.756	ρ = –0.12<br>*P* = 0.549	ρ = 0.21<br>*P* = 0.138	ρ = 0.03<br>*P* = 0.810	ρ = 0.10<br>*P* = 0.461	ρ = –0.08<br>*P* = 0.571	ρ = 0.03<br>*P* = 0.799	ρ = –0.01<br>*P* = 0.907	ρ = 0.06<br>*P* = 0.574	ρ = –0.06<br>*P* = 0.598
Magnitude HTI at ATI start	ρ = –0.06<br>*P* = 0.757	ρ = 0.00<br>*P* = 0.983	ρ = 0.10<br>*P* = 0.585	ρ = –0.09<br>*P* = 0.649	ρ = 0.40<br>*P* = **0.004**	ρ = 0.44<br>*P* = **0.001**	ρ = 0.46<br>*P* = **0.001**	ρ = –0.33<br>*P* = **0.018**	ρ = 0.35<br>*P* = **0.001**	ρ = 0.32<br>*P* = **0.003**	ρ = 0.32<br>*P* = **0.003**	ρ = –0.27<br>*P* = **0.015**
Focus HTI at ATI start	ρ = –0.13<br>*P* = 0.508	ρ = 0.00<br>*P* = 0.988	ρ = 0.02<br>*P* = 0.921	ρ = 0.10<br>*P* = 0.596	ρ = –0.09<br>*P* = 0.509	ρ = 0.12<br>*P* = 0.378	ρ = 0.15<br>*P* = 0.283	ρ = –0.02<br>*P* = 0.915	ρ = 0.07<br>*P* = 0.534	ρ = 0.14<br>*P* = 0.206	ρ = 0.10<br>*P* = 0.375	ρ = –0.02<br>*P* = 0.842
Breadth HTI at ATI start	ρ = –0.10<br>*P* = 0.590	ρ = 0.01<br>*P* = 0.972	ρ = 0.15<br>*P* = 0.420	ρ = –0.15<br>*P* = 0.421	ρ = 0.37<br>*P* = **0.007**	ρ = 0.23<br>*P* = 0.104	ρ = 0.19<br>*P* = 0.168	ρ = –0.23<br>*P* = 0.105	ρ = 0.31<br>*P* = **0.005**	ρ = 0.22<br>*P* = **0.042**	ρ = 0.21<br>*P* = 0.052	ρ = –0.23<br>*P* = **0.041**
Cumulative breadth HTI at ATI start	ρ = –0.06<br>*P* = 0.765	ρ = 0.12<br>*P* = 0.514	ρ = 0.43<br>*P* = **0.016**	ρ = –0.35<br>*P* = 0.058	ρ = 0.25<br>*P* = 0.067	ρ = 0.17<br>*P* = 0.213	ρ = 0.25<br>*P* = 0.070	ρ = –0.29<br>*P* = **0.037**	ρ = 0.22<br>*P* = **0.040**	ρ = 0.23<br>*P* = **0.038**	ρ = 0.29<br>*P* = **0.008**	ρ = –0.31<br>*P* = **0.004**
Peak magnitude HTI	ρ = –0.10<br>*P* = 0.592	ρ = 0.07<br>*P* = 0.717	ρ = 0.30<br>*P* = 0.101	ρ = –0.20<br>*P* = 0.280	ρ = 0.33<br>*P* = **0.016**	ρ = 0.36<br>*P* = **0.007**	ρ = 0.38<br>*P* = **0.004**	ρ = –0.29<br>*P* = **0.032**	ρ = 0.27<br>*P* = **0.012**	ρ = 0.28<br>*P* = **0.009**	ρ = 0.30<br>*P* = **0.005**	ρ = –0.27<br>*P* = **0.012**
Increase from baseline in magnitude HTI at peak	ρ = –0.08<br>*P* = 0.685	ρ = 0.06<br>*P* = 0.739	ρ = 0.29<br>*P* = 0.124	ρ = –0.19<br>*P* = 0.332	ρ = 0.22<br>*P* = 0.121	ρ = 0.28<br>*P* = **0.041**	ρ = 0.30<br>*P* = **0.031**	ρ = –0.26<br>*P* = 0.063	ρ = 0.23<br>*P* = **0.034**	ρ = 0.24<br>*P* = **0.032**	ρ = 0.25<br>*P* = **0.024**	ρ = –0.24<br>*P* = **0.032**

Supplementary Table 3 | Univariate correlate analysis for time off ART. Univariate logistic regression models for time to ART resumption >12 weeks in all participants (vaccine and placebo recipients) from AELIX-002 and AELIX-003 trials who entered the ATI phase (*n* = 88). Odds ratio (OR) and 95% confidence interval (CI) and *P* values are shown. In parentheses, for each variable, the unit of increment is shown for interpretation of the odds ratio. Univariate analyses were not adjusted for multiple comparisons.

**Variable**	**OR (95% CI)**	***P* value**
Treatment allocation (active vs placebo arm)	1.02 (0.42–2.49)	0.9673
Age at study entry (1 year)	1.02 (0.97–1.06)	0.5212
CD4 T cells/mm^3 at study entry (100 cells/mm^3)	1.10 (0.96–1.28)	0.1759
Ratio CD4/CD8 at study entry (0.2 units)	1.16 (0.97–1.42)	0.1253
HIV-1 pVL at ART initiation (1 $\log_{10}$ copies/ml)	0.61 (0.39–0.91)	0.0187
Days from estimated HIV acquisition to ART (7 days)	1.01 (0.94–1.09)	0.7090
Months on ART at study entry (1 month)	1.02 (1.00–1.04)	0.1293
Total proviral HIV DNA at study entry (100 copies/10^6 CD4 T cells)	0.78 (0.62–0.93)	0.0185
Intact proviral HIV DNA at study entry (50 copies/10^6 CD4 T cells)	0.80 (0.58–0.97)	0.0898
Total proviral HIV DNA at ATI start (100 copies/10^6 CD4 T cells)	0.84 (0.67–0.98)	0.0636
Intact proviral HIV DNA at ATI start (50 copies/10^6 CD4 T cells)	0.72 (0.47–0.96)	0.0747
Magnitude HTI at study entry (100 SFC/10^6 PBMC)	1.12 (1.04–1.45)	0.0230
Focus HTI at study entry (10 % HTI/total HIV)	1.05 (0.88–1.24)	0.5997
Breadth HTI at study entry (1 unit)	1.19 (0.91–1.58)	0.2147
Magnitude HTI at ATI start (100 SFC/10^6 PBMC)	1.10 (1.03–1.20)	0.0042
Focus HTI at ATI start (10 % HTI/total HIV)	1.03 (0.89–1.21)	0.6628
Breadth HTI at ATI start (1 unit)	1.28 (1.03–1.62)	0.0325
Cumulative breadth HTI at ATI start (1 unit)	1.23 (1.04–1.47)	0.0225
Peak magnitude HTI (100 SFC/10^6 PBMC)	1.05 (1.01–1.10)	0.0245
Increase magnitude HTI at peak (100 SFC/10^6 PBMC)	1.04 (0.99–1.09)	0.1000

Supplementary Table 4 | Multivariate logistic regression analysis for time off ART.

Multivariate logistic regression model for resuming ART >12 weeks considering covariates in all participants from AELIX-002 and AELIX-003 trials who entered the ATI phase (*n* = 88). Odds ratio (OR) and 95% confidence interval (CI) and *P* values are shown. In parentheses, for each variable, the unit of increment is shown for interpretation of the OR.

	**Full model**		**Final model**	
	**OR (95% CI)**	***P* value**	**OR (95% CI)**	***P* value**
Total proviral HIV DNA at ATI start (100 copies/10^6 CD4 T cells)	0.78 (0.59–0.96)	0.0462	0.79 (0.62–0.95)	0.0316
Magnitude HTI at ATI start (100 SFC/10^6 PBMC)	1.18 (0.99–1.45)	0.0872	1.13 (1.05–1.23)	0.0020
pVL at ART initiation (1 log_{10} copies/ml)	0.67 (0.39–1.08)	0.1166		
Months on ART at study entry (1 month)	1.02 (0.99–1.05)	0.1631		

Supplementary Note | List of AELIX-002 and AELIX-003 Study Group members

<u>**AELIX-002 Study Group members**</u>

From Fundació Lluita contra les Infeccions, Deaprtment of Infectious Diseases, Hospital Universitari Germans Trias i Pujol, Badalona, Spain: Yovaninna Alarcón-Soto, Lucía Bailón, Susana Benet, Patricia Cobarsí, Roser Escrig, Silvia Gel, Cora Loste, Miriam López, Cristina Martinez, Laura Mas, Cristina Miranda, José Moltó, Jose Muñoz, Aroa Nieto, Helena Pera, Francisco Perez, Jordi Puig, Lara Teruel, Albert Tuldrà and Jessica Toro.

From IrsiCaixa, Hospital Universitari Germans Trias i Pujol, Badalona, Spain: Christian Brander, Maria Casadellà, Samandhy Cedeño, Bonaventura Clotet, Josep Coll, Tuixent Escribà, Anuska Llano, Mireia Manent, Chiara Mancuso, Beatriz Mothe, Marc Noguera-Julian, Roger Paredes, Mariona Parera, Miriam Rosás-Umbert, Marta Ruiz-Riol and Bruna Oriol-Tordera.

From Projecte dels Noms-Hispanosida, BCN Checkpoint, Barcelona, Spain: Javier Fernández, Michael Meulbroek, Félix Perez, Ferran Pujol, Angel Rivero and Jorge Saz.

From AELIX Therapeutics S.L., Barcelona, Spain: Lance Berman, Jose Luís Cabero, Margarida Garcia, Anne R. Leselbaum, Marc Mansour, Ian McGowan and Jordi Naval.

From the Jenner Institute, The Nuffield Department of Medicine, University of Oxford, UK: Tomáš Hanke and Edmund G. Wee.

From Gilead Sciences, Foster City, US: Devi SenGupta and Romas Geleziunas.

From Germans Trias i Pujol Research Institute, Badalona, Spain: Ana María Barriocanal

<u>**AELIX-003 Study Group members**</u>

From Fundació Lluita contra les Infeccions, Department of Infectious Diseases, Hospital Universitari Germans Trias i Pujol, Badalona, Spain: Yovaninna Alarcón-Soto, Lucia

Bailón, Susana Benet, Patricia Cobarsí, Cristina Martinez, José Moltó, Aroa Nieto, Francisco Perez and Jordi Puig.

From IrsiCaixa, Hospital Universitari Germans Trias i Pujol, Badalona, Spain: Christian Brander, Samandhy Cedeño, Bonaventura Clotet, Eulalia Grau, Anuska Llano, Beatriz Mothe and Roger Paredes.

From AELIX Therapeutics S.L., Barcelona, Spain: Alvaro Aranguen, Jose Luís Cabero, Margarida Garcia-Garcia, Isabel Leal, Ian McGowan and Jordi Naval.

From Gilead Sciences, Foster City, CA, USA: Yanhui Cai, Romas Geleziunas, Susan Guo, Devi SenGupta, Elena Vendrame and Jeffrey J. Wallin

From Hospital Universitari Vall d´Hebron, Barcelona, Spain: Adrian Curran, Vicenç Falcó, Jordi Navarro, Bibiana Planas, Paula Suanzes, Joaquin Burgos, María José Buzón, Meritxell Genescà and Judith Grau.

From Hospital Universitario La Paz, Madrid, Spain: Jose Ramón Arribas, Alberto Borobia, Carmen Busca, Julen Cadiñanos, Juan González García, Victor Hontañon, Javier Queiruga, Rafael Mican and Enrique Seco.

From Hospital General Universitario Gregorio Marañón, Madrid, Spain: Juan Berenguer, Cristina Diez, Juan Carlos Lopez Bernaldo de Quirós, Paloma Gijón, Leire Pérez-Latorre and Margarita Ramirez.

From Hospital Universitario La Princesa, Madrid, Spain: Lucio Jesús García-Fraile, Samuel Martín Vílchez, Gina Mejía-Abril, Alejandro de Miguel Cáceres, Ignacio de los Santos, José María Serra and Tamara De la Torre Muñoz.

From Hospital Clinic de Barcelona, Barcelona, Spain: Juan Ambrosioni, Eva Ariza and Jose Maria Miró.

From Hospital Universitari de Belltvige, Barcelona, Spain: Anna Ferrer, Benito Garcia, Arkaitz Imaz, Sandra Morenilla, Jordi Niubó, Camila Piatti, Sofía Scévola, Irene Soriano and Daniel Vázquez.

From Hospital Universitari Ramón y Cajal, Madrid, Spain: Santiago Moreno.

From Hospital Santa Creu i Sant Pau, Barcelona, Spain: Pere Domingo
